# Lethal form of a late-onset aquaporin-4 antibody-positive NMOSD related to the immune checkpoint inhibitor nivolumab

**DOI:** 10.1007/s00415-021-10913-y

**Published:** 2021-11-28

**Authors:** David Weiss, Daniel Cantré, Uwe K. Zettl, Alexander Storch, Johannes Prudlo

**Affiliations:** 1 Department of Neurology, Rostock University Medical Center, Rostock, Germany; 2 Institute of Diagnostic and Interventional Radiology, Pediatric Radiology and Neuroradiology, Rostock University Medical Center, Rostock, Germany; 3 Neuroimmunological Section, Department of Neurology, Rostock University Medical Center, Gehlsheimer Str. 20, 18147 Rostock, Germany; 4 German Center for Neurodegenerative Diseases (DZNE), Rostock, Germany

Dear Sirs,

Immune checkpoint inhibitors (ICIs) are implemented to achieve high antitumor immunity. One of the costs of these advances is the emergence of a new spectrum of immune-related adverse events (irAEs) [1]. Although high-grade neurological immune-related adverse events, such as longitudinal extensive transverse myelitis (LETM) are rare, they are being increasingly reported and associated with poor recovery [2]. With only two reported cases, ICI-associated AQP4 antibody (Ab)-positive NMOSD is exceedingly rare: One previously reported case was a patient with lung carcinoma, who developed a steroid refractory AQP4-Ab-positive late-onset NMOSD (LO-NMOSD) after treatment with the programmed cell death 1 checkpoint inhibitor (PD-1) nivolumab and responded poorly to plasma exchange [3]. Another reported case describes an AQP4-Ab-positive LO-NMOSD in a patient with lung adenocarcinoma after receiving the first cycle of the anti-PD-1 checkpoint inhibitor pembrolizumab [4].

The case presented here describes a fatal AQP4-Ab-positive LO-NMOSD in an 81-year-old Caucasian female patient with clear cell renal cell carcinoma after treatment with nivolumab. This case raises the question of the pathophysiological role of AQP4-Ab in a paraneoplastic context under treatment with ICI and, thus, has implications for ICI treatment.

16 months prior to admission, the patient was diagnosed with clear cell renal cell carcinoma (pT3a pNx V0 L0 Pn0 R0 G2), and nephrectomy was performed. Five months later, pulmonary metastases were diagnosed and 10 cycles of the PD-1 inhibitor nivolumab were administered, resulting in stable disease. Two weeks prior to admission, oral prednisone was administered for treatment of nivolumab-related pneumonitis.

**Fig. 1:**
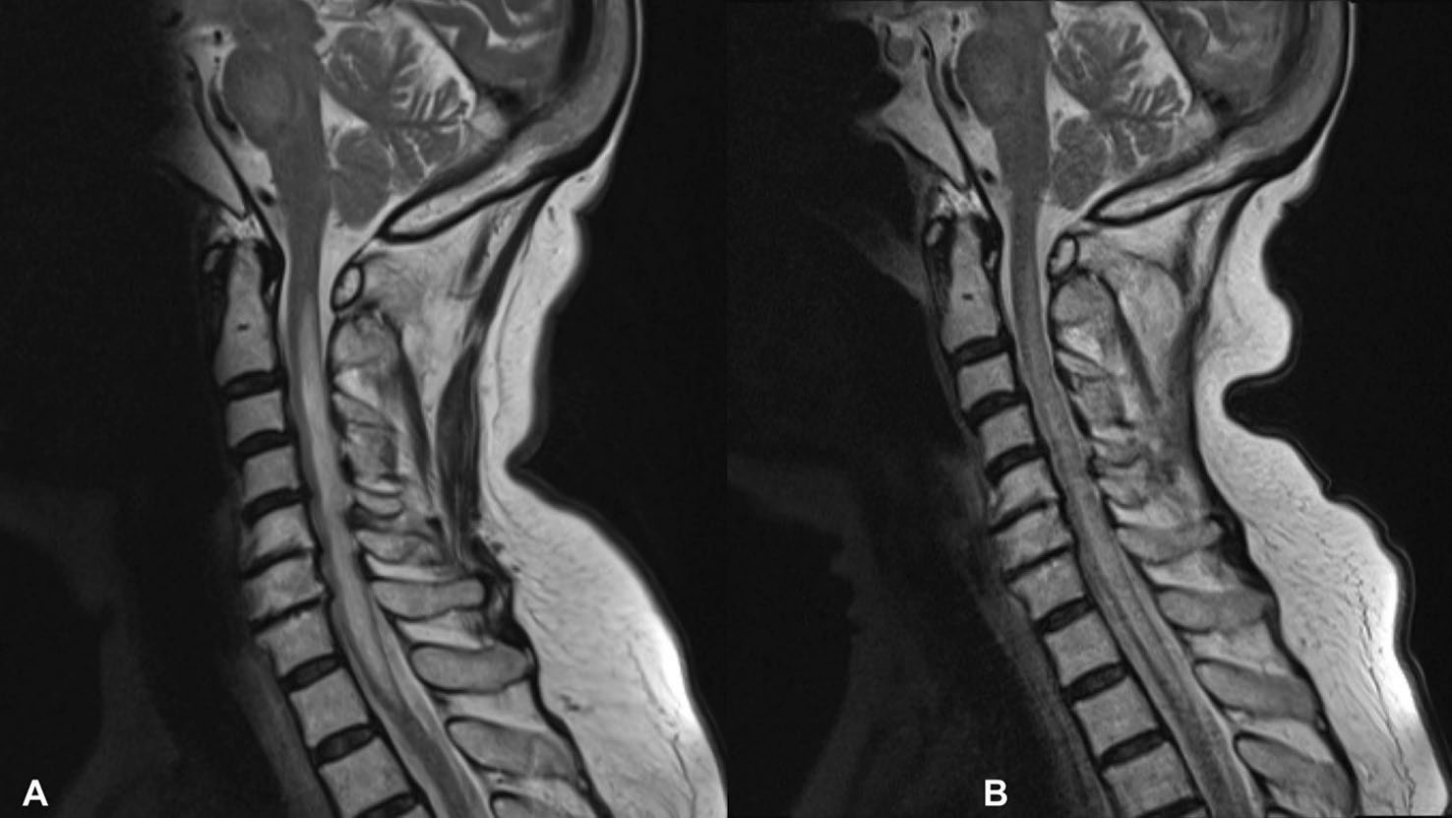
Sagittal T2-weighted MRI at 1.5T demonstrating the LETM extending from the C1 to T1 level on admission (**A**) and longitudinal progression 7 days later despite therapy, reaching the level of the lower vertebral body endplate of T2 (**B**)

On admission, the patient demonstrated a central cord syndrome, bilateral pyramidal signs, and sensory loss in a cape distribution, with loss of pain and temperature in the arms, shoulders, and upper chest. Symptom onset was 5 days prior to admission, with paresthesia in both upper arms, spreading downwards to the forearms. There was a sensory level at the C5 dermatome and posterior column sensory involvement of the upper limbs. Reflexes were absent in the upper limbs but brisk in the lower limbs. No respiratory involvement and no visual impairment could be detected. Spine MRI revealed LETM with centrally located T2-hyperintensity and cord swelling from the C1 to T1 level (Fig. 1), but no gadolinium enhancement. The cerebrospinal fluid (CSF) showed neutrophilic pleocytosis at 65/mL and elevated total protein levels of 63 mg/dl, but no CSF-restricted oligoclonal bands. Neither malignant cells nor infectious agents were detected. An investigation for makers of autoimmune disorders, anti-neuronal antibodies, and myelin-oligodendrocyte glycoprotein (MOG) antibodies yielded negative results. In contrast, AQP4-Ab tested positive (titer 1:100, normal < 1:10). Antibody status prior to our investigation was not available.

A nivolumab-induced LETM was suspected, and high-dose intravenous methylprednisolone was administered, synchronously with 5 cycles of plasma exchange. However, despite aggressive treatment, fulminant clinical worsening was observed, with rapid progression to tetraplegia with respiratory involvement without the need for mechanical ventilation, paralleled by radiologically progressive myelitis expanding downwards to T2 in repeat spinal MRI (Fig. 1). 14 days following the onset of spinal cord syndrome, the patient died due to rapid disease progression and severe respiratory failure.

This case describes a lethal nivolumab-associated AQP4-Ab-positive LO-NMOSD in a paraneoplastic context. AQP4 is overexpressed in renal and lung cancer [5]. Therefore, we hypothesize that Ab production against AQP4 in our case is a misdirected antitumor response, facilitated and amplified by ICI-/T cell-mediated excessive cellular and humoral immunity, thus constituting both a paraneoplastic phenomenon and irAE. It has been demonstrated that in other autoimmune diseases, such as myasthenia gravis, the disease course is more severe when ICI-related [6]. Likewise, our case indicates that ICI-associated AQP4-Ab-positive LO-NMOSD possibly has a more fulminant and therapy-refractory course than non-ICI-associated forms.

One possible explanation for this is the excessive production of potentially autoreactive T and B cells as a consequence of the ICI-induced interruption of regulatory T cell (Tregs) functionality: the negative immunomodulatory PD-1 pathway plays a pivotal regulatory role in the activation of Tregs, which both regulate B cell differentiation into antibody-producing plasma cells and suppress autoreactive T cells [1]. By blocking the PD-1 pathway, excessive B cell activation with secretion of AQP4-Abs, and an abnormal activation of autoreactive T cells occur.

In conclusion, our case strongly suggests that AQP4-Ab-positive LO-NMOSD, inherently associated with a worse outcome [7], can occur as a paraneoplastic phenomenon that can be triggered and/or exacerbated by ICIs as a result of excessive immune activation. Whether AQP4 antibody screening should be performed routinely prior to ICI initiation in addition to whether ICIs should be avoided when AQP4-Abs have been detected may deserve more serious and urgent consideration. Given the foudroyant disease course, administering monoclonal antibody therapy, such as Rituximab, should be considered early.
